# Crystal structure of (2′,3,6′-tri­chloro­biphenyl-2-yl)boronic acid tetra­hydro­furan monosolvate

**DOI:** 10.1107/S205698901502054X

**Published:** 2015-11-07

**Authors:** Krzysztof Durka, Tomasz Kliś, Janusz Serwatowski

**Affiliations:** a Physical Chemistry Department, Faculty of Chemistry, Warsaw University of Technology, Noakowskiego 3, 00-664 Warsaw, Poland

**Keywords:** aryl­boronic acid, hydrogen-bonding inter­actions, halogen-bonding inter­actions, biphen­yl, THF solvate, crystal structure

## Abstract

The title compound, C_12_H_8_BCl_3_O_2_·C_4_H_8_O, crystallizes as a tetra­hydro­furan monosolvate. The boronic acid group adopts a *syn–anti* conformation and is significantly twisted along the carbon–boron bond by 69.2 (1)°, due to considerable steric hindrance from the 2′,6′-di­chloro­phenyl group that is located *ortho* to the boronic acid substituent. The phenyl rings of the biphenyl are almost perpendicular to one another, with a dihedral angle of 87.9 (1)° between them. In the crystal, adjacent mol­ecules are linked *via* O—H⋯O inter­actions to form centrosymmetric dimers with *R*_2_^2^(8) motifs, which have recently been shown to be energetically very favourable. The hy­droxy groups are in an *anti* conformation and are also engaged in hydrogen-bonding inter­actions with the O atom of the tetra­hydro­furan solvent mol­ecule. Cl⋯Cl halogen-bonding inter­actions [Cl⋯Cl = 3.464 (1) Å] link neigbouring dimers into chains running along [010]. Further aggregation occurs due to an additional Cl⋯Cl halogen bond [Cl⋯Cl = 3.387 (1) Å].

## Abstract 2

In this manuscript the mol­ecular and supra­molecular structure of 2′,3,6′-tri­chloro­biphenyl-2-ylboronic acid tetra­hydro­furan monosolvate is presented

## Chemical context   

**Figure 1:**
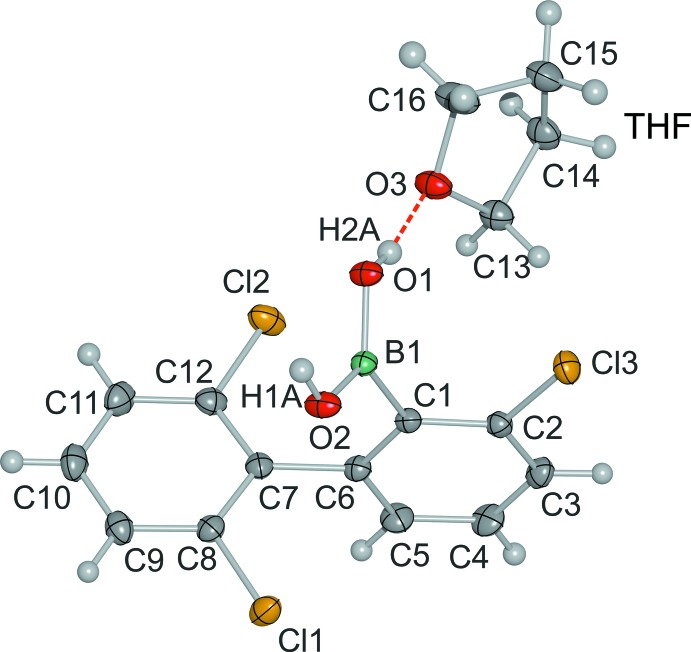
The structure of **1**, showing the atom numbering, with displacement ellipsoids drawn at the 50% probability level.

Boronic acids and their derivatives have been studied intensively in recent years due to their numerous applications in organic, analytical and materials chemistry (Hall, 2011 ▸; Furukawa & Yaghi, 2009 ▸). They are widely used in medicine, for example, as anti­fungal and anti­bacterical agents (Adamczyk-Woźniak *et al.*, 2015 ▸; Kane *et al.*, 2003 ▸; Vogt *et al.*, 2013 ▸). Besides these applications, phenyl­boronic acids have also been studied in terms of crystal engineering (Nishiyabu *et al.*, 2011 ▸; Severin, 2009 ▸). In contrast, biphenyl-based boronic acids have been largely neglected. Exceptions to this include reports of the crystal structures of (2-bi­phenyl­yl)boronic acid (Filthaus *et al.*, 2008 ▸) and (2-meth­oxy-3-biphen­yl)boronic acid (Davies *et al.*, 2008 ▸). In this manuscript we focus our attention on a sterically hindered boronic acid derivative based on a biphenyl core with a boronic group located at the 2-position of one benzene ring with a Cl substituent at the 3-position. The second benzene ring of the biphenyl ring system carries chlorine substituents at the 2- and 6-positions. This mol­ecule crystallized as a 1:1 solvate with THF, Fig. 1 ▸.
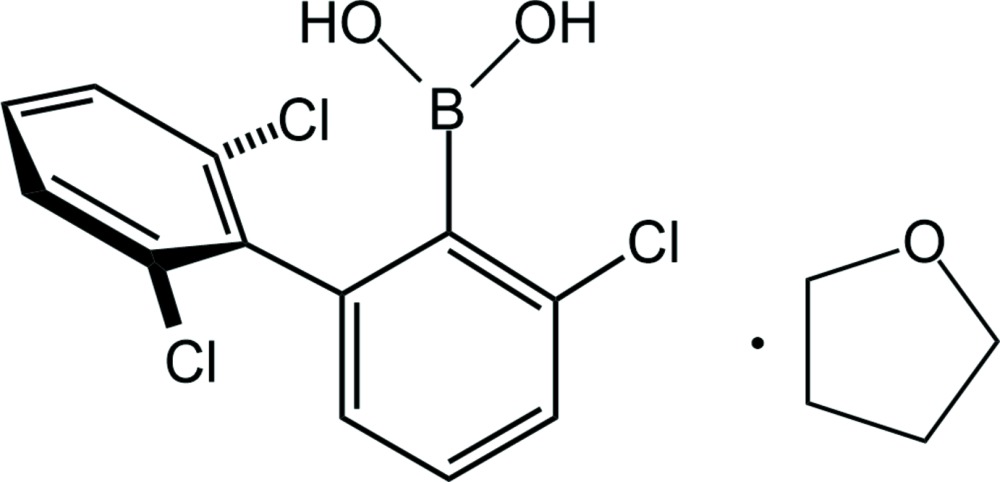


## Structural commentary   

The B—C [1.5907 (16) Å] and B—O [1.3514 (14), 1.3641 (14) Å] bonds in the title compound (I) are within the expected range typically observed for boronic acids (Madura *et al.*, 2014 ▸; Luliński *et al.*, 2007 ▸; Maly *et al.*, 2006 ▸; Shimpi *et al.*, 2007 ▸; Durka *et al.*, 2012 ▸). The mol­ecular structure shows that the B(OH)_2_ group adopts the usual *syn–anti* conformation (Fig. 1 ▸). The boronic acid substituent is significantly rotated about the C—B bond in order to minimize the steric hindrance between the boronic group and the adjacent 2′,6′-di­chloro­phenyl ring [*τ*_C2—C1—B1—O1_ = 69.2 (2)°]. In the structure of the related (2-bi­phenyl­yl)boronic acid (Filthaus *et al.*, 2008 ▸) this torsion angle is some 20° smaller, which clearly shows the influence of the three chlorine substituents on this structure. It is also notable that in (I) the phenyl rings of the biphenyl system are almost perpendicular to one another [*τ*_C1—C6—C7—C11_ = 87.9 (1)°], whereas in (2-bi­phenyl­yl)boronic acid they are rotated by only 48.4 or 45.4° for the two unique mol­ecules in the asymmetric unit.

## Supra­molecular features   

**Figure 2:**
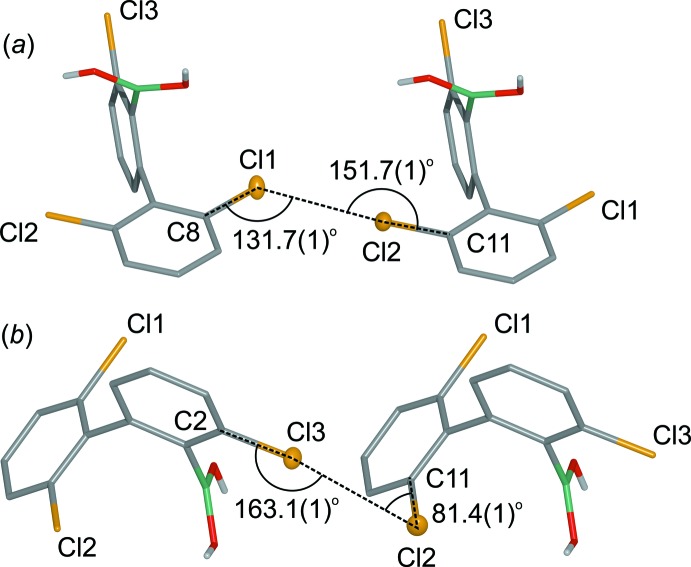
Type I (*a*) and II (*b*) Cl⋯Cl halogen bonds in (I).

**Figure 3:**
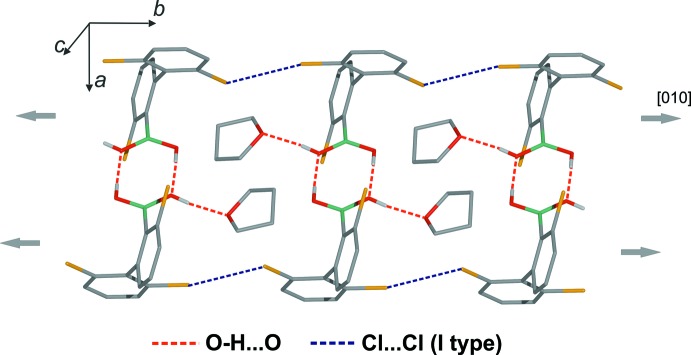
Mol­ecular chains formed along *b* by type 1 Cl⋯Cl halogen bonds. Also shown are inversion dimers and inclusion of the solvent through O—H⋯O hydrogen bonds. Aromatic H atoms have been omitted for clarity.

**Figure 4:**
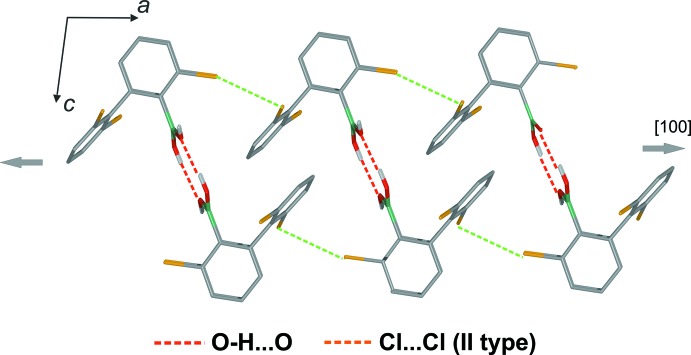
Crystal packing showing inter­molecular O—H⋯O and type II Cl⋯Cl inter­actions.

In the crystal, centrosymmetric O—H⋯O hydrogen-bonded dimers are formed. The *anti*-oriented OH group is engaged in an inter­molecular O—H⋯O hydrogen bond (Table 1 ▸) with the oxygen atom from the tetra­hydro­furan solvate mol­ecule. Because all of the hydrogen-bond acceptor centres are saturated, the *syn* OH group is not involved in any side hydrogen-bond inter­actions. Neighbouring dimers are connected through Cl⋯Cl halogen bonds [*d*_Cl⋯Cl_ = 3.464 (1) Å; the sum of the van der Waals radii for Cl is 3.50 Å]. In terms of geometry of this contact, it can be classified as a type I halogen bond (Fig. 2 ▸ *a*), (Metrangolo *et al.*, 2005 ▸; Nayak *et al.*, 2011 ▸). These contacts result in the formation of mol­ecular chains propagating along [010] (Fig. 3 ▸). A three-dimensional network forms through additional Cl⋯Cl halogen bonds (Fig. 4 ▸) of type II [*d*_Cl⋯Cl_ = 3.387 (1) Å] (Fig. 2 ▸ *b*).

## Synthesis and crystallization   

**Figure 5:**
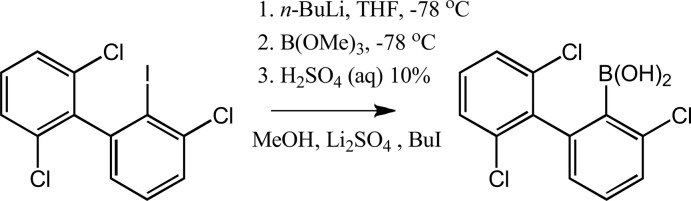
The synthesis of 2-chloro-6-(2′,6′-di­chloro­phen­yl)phenyl­boronic acid.

**Synthesis of (I) (Fig. 5 ▸):** A solution of 2-iodo-2′,3,6′-tri­chloro­biphenyl (3.8 g, 10 mmol) in THF (50 mL) was added to a stirred solution of *n*-BuLi (10 mmol) in THF (30 mL) at 195 K. The resulting colorless solution was stirred for 1 h to give a colorless precipitate. The electrophile, B(OMe)_3_ (2.1 g, 20 mmol) was then added to the stirred mixture to give a colorless solution which was stirred for 1 h and then hydrolyzed with H_2_O (100 mL). Dilute aq. H_2_SO_4_ was added until the pH was slightly acidic. Et_2_O (50 mL) was next added and the mixture stirred for 10 min. The organic phase was separ­ated and the aqueous phase was extracted with Et_2_O (20 mL). The combined organic solutions were dried over MgSO_4_ and evaporated to give a colorless precipitate, yield 2.0 g (66%). ^1^H NMR (400 MHz, acetone-*d*_6_): δ = 8.00 (2H, *s*, OH), 7.51 (2H, *m*), 7.39 (3H, *m*), 7.05 (1H, m*);* ^13^C{1H} NMR (100.6 MHz, acetone-*d*_6_): δ = 141.49, 139.62, 139 (*br*), 136.17, 134.84, 130.49, 129.99, 128.24, 128.14, 127.53.

Crystals suitable for X-ray diffraction analysis were grown by slow evaporation of a THF solution.

## Refinement details   

Crystal data, data collection and structure refinement details are summarized in Table 2 ▸. All CH hydrogen atoms were placed in calculated positions with C—H distances of 0.95 or 0.99 Å. They were included in the refinement in the riding-motion approximation with *U*_iso_(phenyl H) = 1.2*U*_eq_(C). The positions of the OH hydrogen atoms were first found in a difference map. Then their bond lengths were restrained in the last least-squares cycles, with an O—H distance of 0.85 Å and their coordinates refined with *U*_iso_(hydroxyl H) = 1.5*U*_eq_(O).

## Supplementary Material

Crystal structure: contains datablock(s) I. DOI: 10.1107/S205698901502054X/sj5482sup1.cif

Structure factors: contains datablock(s) I. DOI: 10.1107/S205698901502054X/sj5482Isup2.hkl

Supporting information file. DOI: 10.1107/S205698901502054X/sj5482Isup3.cdx

Supporting information file. DOI: 10.1107/S205698901502054X/sj5482Isup4.cdx

Supporting information file. DOI: 10.1107/S205698901502054X/sj5482Isup5.cml

CCDC reference: 1434205

Additional supporting information:  crystallographic information; 3D view; checkCIF report

## Figures and Tables

**Table 1:** Hydrogen-bond geometry (Å, °)

*D*—H⋯*A*	*D*—H	H⋯*A*	*D*⋯*A*	*D*—H⋯*A*
O1—H1*A*⋯O3	0.82 (1)	1.84 (1)	2.6475 (12)	169 (2)
O2—H2*A*⋯O1^i^	0.84 (1)	1.96 (1)	2.7997 (12)	175 (2)

Symmetry code: (i) 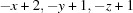.

**Table 2:** Experimental details

Crystal data	
Chemical formula	C_12_H_8_BCl_3_O_2_·C_4_H_8_O
*M*_r_	373.45
Crystal system, space group	Triclinic, *P* 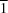
Temperature (K)	130
*a*, *b*, *c* (Å)	8.3306 (3), 8.7122 (2), 12.4307 (4)
α, β, γ (°)	98.683 (3), 97.737 (3), 99.398 (3)
*V* (Å^3^)	868.07 (5)
*Z*	2
Radiation type	Mo *K*α
μ (mm^−1^)	0.54
Crystal size (mm)	0.15 × 0.12 × 0.10

Data collection	
Diffractometer	Agilent SuperNova Dual Source diffractometer with an Atlas detector
Absorption correction	Multi-scan (*CrysAlis PRO*; Agilent, 2014 ▸)
*T*_min_, *T*_max_	0.795, 1.000
No. of measured, independent and observed [*I* > 2σ(*I*)] reflections	28647, 6107, 5162
*R*_int_	0.032
(sin θ/λ)_max_ (Å^−1^)	0.758

Refinement	
*R*[*F* ^2^ > 2σ(*F* ^2^)], *wR*(*F* ^2^), *S*	0.034, 0.085, 1.05
No. of reflections	6107
No. of parameters	214
No. of restraints	2
H-atom treatment	H atoms treated by a mixture of independent and constrained refinement
Δρ_max_, Δρ_min_ (e Å^−3^)	0.45, −0.36

Computer programs: *CrysAlis PRO* (Agilent, 2014 ▸), *SHELXS97* (Sheldrick, 2015 ▸), *SHELXL2013* (Sheldrick, 2015 ▸), *DIAMOND* (Brandenburg, 2005 ▸) and *publCIF* (Westrip, 2010 ▸).

## supplementary crystallographic information

### Crystal data

**Table:**

C_12_H_8_BCl_3_O_2_·C_4_H_8_O	*F*(000) = 384
*M**_r_* = 373.45	*D*_x_ = 1.429 Mg m^−^^3^
Triclinic, *P*1	Melting point: 411 K
*a* = 8.3306 (3) Å	Mo *K*α radiation, λ = 0.71073 Å
*b* = 8.7122 (2) Å	Cell parameters from 14113 reflections
*c* = 12.4307 (4) Å	θ = 2.4–32.4°
α = 98.683 (3)°	µ = 0.54 mm^−^^1^
β = 97.737 (3)°	*T* = 130 K
γ = 99.398 (3)°	Fragment, colourless
*V* = 868.07 (5) Å^3^	0.15 × 0.12 × 0.10 mm
*Z* = 2	

### Data collection

**Table:**

Agilent SuperNova Dual Source diffractometer with an Atlas detector	6107 independent reflections
Radiation source: SuperNova (Mo) X-ray Source	5162 reflections with *I* > 2σ(*I*)
Mirror monochromator	*R*_int_ = 0.032
Detector resolution: 5.2195 pixels mm^-1^	θ_max_ = 32.6°, θ_min_ = 2.4°
ω scans	*h* = −12→12
Absorption correction: multi-scan (*CrysAlis PRO*; Agilent, 2014)	*k* = −13→13
*T*_min_ = 0.795, *T*_max_ = 1.000	*l* = −18→18
28647 measured reflections	

### Refinement

**Table:**

Refinement on *F*^2^	2 restraints
Least-squares matrix: full	Hydrogen site location: mixed
*R*[*F*^2^ > 2σ(*F*^2^)] = 0.034	H atoms treated by a mixture of independent and constrained refinement
*wR*(*F*^2^) = 0.085	*w* = 1/[σ^2^(*F*_o_^2^) + (0.0328*P*)^2^ + 0.3549*P*] where *P* = (*F*_o_^2^ + 2*F*_c_^2^)/3
*S* = 1.05	(Δ/σ)_max_ = 0.001
6107 reflections	Δρ_max_ = 0.45 e Å^−^^3^
214 parameters	Δρ_min_ = −0.36 e Å^−^^3^

### Special details

**Table:**

Experimental. Absorption correction: CrysAlisPro (Agilent Technologies, 2014), Empirical absorption correction using spherical harmonics, implemented in SCALE3 ABSPACK scaling algorithm.
Geometry. All e.s.d.'s (except the e.s.d. in the dihedral angle between two l.s. planes) are estimated using the full covariance matrix. The cell e.s.d.'s are taken into account individually in the estimation of e.s.d.'s in distances, angles and torsion angles; correlations between e.s.d.'s in cell parameters are only used when they are defined by crystal symmetry. An approximate (isotropic) treatment of cell e.s.d.'s is used for estimating e.s.d.'s involving l.s. planes.

### Fractional atomic coordinates and isotropic or equivalent isotropic displacement parameters (Å^2^)

**Table:**

	*x*	*y*	*z*	*U*_iso_*/*U*_eq_	
C1	0.77416 (13)	0.33187 (12)	0.22248 (8)	0.01544 (18)	
C2	0.85513 (13)	0.33777 (12)	0.13113 (9)	0.01761 (19)	
C3	0.77605 (15)	0.28436 (14)	0.02290 (9)	0.0221 (2)	
H3	0.8355	0.2914	−0.0368	0.027*	
C5	0.52304 (15)	0.21255 (15)	0.09167 (10)	0.0233 (2)	
H5	0.4085	0.1690	0.0782	0.028*	
C6	0.60414 (13)	0.26808 (13)	0.19972 (9)	0.01703 (19)	
C7	0.50529 (13)	0.26201 (13)	0.29144 (9)	0.01742 (19)	
C8	0.47656 (14)	0.12861 (13)	0.34104 (9)	0.0188 (2)	
C9	0.38144 (14)	0.12012 (15)	0.42447 (10)	0.0230 (2)	
H9	0.3646	0.0276	0.4565	0.028*	
C10	0.31167 (15)	0.24852 (16)	0.46023 (11)	0.0263 (2)	
H10	0.2466	0.2441	0.5172	0.032*	
C11	0.43238 (14)	0.38827 (13)	0.33025 (10)	0.0211 (2)	
C12	0.33610 (15)	0.38351 (16)	0.41341 (11)	0.0262 (2)	
H12	0.2879	0.4714	0.4377	0.031*	
C13	0.60897 (16)	0.22065 (15)	0.00366 (10)	0.0257 (2)	
H13	0.5531	0.1825	−0.0697	0.031*	
C14	0.82695 (16)	0.75012 (14)	0.14313 (10)	0.0241 (2)	
H14A	0.7067	0.7154	0.1184	0.029*	
H14B	0.8843	0.6702	0.1077	0.029*	
C15	0.88574 (17)	0.91105 (15)	0.11452 (11)	0.0283 (3)	
H15A	0.8014	0.9785	0.1202	0.034*	
H15B	0.9142	0.9012	0.0393	0.034*	
C16	1.03885 (18)	0.97668 (16)	0.20241 (12)	0.0325 (3)	
H16A	1.1369	0.9378	0.1804	0.039*	
H16B	1.0621	1.0937	0.2165	0.039*	
C17	0.9899 (2)	0.91251 (17)	0.30259 (12)	0.0358 (3)	
H17A	1.0868	0.8873	0.3471	0.043*	
H17B	0.9443	0.9914	0.3495	0.043*	
B1	0.86796 (14)	0.39672 (14)	0.34516 (10)	0.0160 (2)	
O1	0.92407 (11)	0.55444 (9)	0.38238 (7)	0.02050 (16)	
O2	0.89019 (11)	0.29285 (10)	0.41379 (7)	0.02293 (17)	
O3	0.86646 (12)	0.77103 (10)	0.26144 (7)	0.02631 (19)	
Cl1	0.56394 (4)	−0.03377 (3)	0.29838 (2)	0.02648 (7)	
Cl2	0.46307 (4)	0.55927 (3)	0.27352 (3)	0.02936 (8)	
Cl3	1.06696 (3)	0.41387 (4)	0.15296 (2)	0.02402 (7)	
H1A	0.902 (2)	0.6121 (19)	0.3383 (13)	0.036*	
H2A	0.941 (2)	0.336 (2)	0.4767 (12)	0.036*	

### Atomic displacement parameters (Å^2^)

**Table:**

	*U*^11^	*U*^22^	*U*^33^	*U*^12^	*U*^13^	*U*^23^
C1	0.0179 (5)	0.0142 (4)	0.0137 (4)	0.0028 (3)	0.0005 (3)	0.0028 (3)
C2	0.0196 (5)	0.0163 (4)	0.0168 (5)	0.0029 (4)	0.0027 (4)	0.0034 (4)
C3	0.0289 (6)	0.0237 (5)	0.0148 (5)	0.0061 (4)	0.0047 (4)	0.0040 (4)
C5	0.0212 (5)	0.0264 (6)	0.0181 (5)	0.0002 (4)	−0.0030 (4)	0.0013 (4)
C6	0.0186 (5)	0.0169 (4)	0.0146 (4)	0.0025 (4)	0.0002 (4)	0.0028 (4)
C7	0.0156 (4)	0.0194 (5)	0.0152 (4)	0.0009 (4)	0.0001 (3)	0.0015 (4)
C8	0.0197 (5)	0.0202 (5)	0.0150 (5)	0.0025 (4)	0.0003 (4)	0.0014 (4)
C9	0.0208 (5)	0.0284 (6)	0.0192 (5)	0.0013 (4)	0.0022 (4)	0.0065 (4)
C10	0.0186 (5)	0.0363 (7)	0.0236 (6)	0.0033 (5)	0.0060 (4)	0.0044 (5)
C11	0.0176 (5)	0.0196 (5)	0.0243 (5)	0.0016 (4)	0.0010 (4)	0.0027 (4)
C12	0.0193 (5)	0.0296 (6)	0.0293 (6)	0.0065 (4)	0.0056 (4)	0.0005 (5)
C13	0.0305 (6)	0.0290 (6)	0.0138 (5)	0.0024 (5)	−0.0024 (4)	0.0013 (4)
C14	0.0267 (6)	0.0221 (5)	0.0226 (5)	0.0031 (4)	0.0000 (4)	0.0059 (4)
C15	0.0332 (7)	0.0258 (6)	0.0278 (6)	0.0044 (5)	0.0057 (5)	0.0114 (5)
C16	0.0333 (7)	0.0243 (6)	0.0375 (7)	−0.0023 (5)	0.0049 (6)	0.0068 (5)
C17	0.0411 (8)	0.0261 (6)	0.0328 (7)	−0.0066 (5)	−0.0064 (6)	0.0072 (5)
B1	0.0152 (5)	0.0170 (5)	0.0146 (5)	0.0022 (4)	0.0009 (4)	0.0015 (4)
O1	0.0278 (4)	0.0162 (4)	0.0149 (4)	0.0019 (3)	−0.0030 (3)	0.0027 (3)
O2	0.0302 (4)	0.0179 (4)	0.0170 (4)	0.0010 (3)	−0.0053 (3)	0.0036 (3)
O3	0.0328 (5)	0.0202 (4)	0.0233 (4)	−0.0005 (3)	−0.0017 (3)	0.0072 (3)
Cl1	0.04097 (17)	0.02093 (13)	0.02001 (13)	0.00986 (11)	0.00736 (11)	0.00481 (10)
Cl2	0.02627 (15)	0.02037 (13)	0.04284 (18)	0.00462 (10)	0.00663 (12)	0.00884 (12)
Cl3	0.01988 (13)	0.02816 (14)	0.02462 (14)	0.00239 (10)	0.00640 (10)	0.00636 (11)

### Geometric parameters (Å, º)

**Table:**

C1—C2	1.3999 (15)	C11—Cl2	1.7388 (12)
C1—C6	1.4081 (15)	C12—H12	0.9500
C1—B1	1.5907 (16)	C13—H13	0.9500
C2—C3	1.3902 (16)	C14—O3	1.4400 (15)
C2—Cl3	1.7498 (11)	C14—C15	1.5183 (17)
C3—C13	1.3859 (18)	C14—H14A	0.9900
C3—H3	0.9500	C14—H14B	0.9900
C5—C13	1.3900 (17)	C15—C16	1.532 (2)
C5—C6	1.3954 (15)	C15—H15A	0.9900
C5—H5	0.9500	C15—H15B	0.9900
C6—C7	1.4959 (15)	C16—C17	1.517 (2)
C7—C11	1.3959 (16)	C16—H16A	0.9900
C7—C8	1.3976 (15)	C16—H16B	0.9900
C8—C9	1.3908 (16)	C17—O3	1.4465 (16)
C8—Cl1	1.7395 (12)	C17—H17A	0.9900
C9—C10	1.3851 (18)	C17—H17B	0.9900
C9—H9	0.9500	B1—O2	1.3514 (14)
C10—C12	1.3869 (19)	B1—O1	1.3641 (14)
C10—H10	0.9500	O1—H1A	0.821 (14)
C11—C12	1.3924 (17)	O2—H2A	0.838 (14)
			
C2—C1—C6	116.31 (9)	C3—C13—C5	120.03 (11)
C2—C1—B1	121.95 (9)	C3—C13—H13	120.0
C6—C1—B1	121.73 (9)	C5—C13—H13	120.0
C3—C2—C1	123.29 (10)	O3—C14—C15	105.36 (10)
C3—C2—Cl3	117.77 (9)	O3—C14—H14A	110.7
C1—C2—Cl3	118.93 (8)	C15—C14—H14A	110.7
C13—C3—C2	118.82 (11)	O3—C14—H14B	110.7
C13—C3—H3	120.6	C15—C14—H14B	110.7
C2—C3—H3	120.6	H14A—C14—H14B	108.8
C13—C5—C6	120.36 (11)	C14—C15—C16	102.13 (10)
C13—C5—H5	119.8	C14—C15—H15A	111.3
C6—C5—H5	119.8	C16—C15—H15A	111.3
C5—C6—C1	121.18 (10)	C14—C15—H15B	111.3
C5—C6—C7	118.41 (10)	C16—C15—H15B	111.3
C1—C6—C7	120.40 (9)	H15A—C15—H15B	109.2
C11—C7—C8	116.15 (10)	C17—C16—C15	102.58 (11)
C11—C7—C6	121.66 (10)	C17—C16—H16A	111.3
C8—C7—C6	122.17 (10)	C15—C16—H16A	111.3
C9—C8—C7	122.68 (11)	C17—C16—H16B	111.3
C9—C8—Cl1	118.09 (9)	C15—C16—H16B	111.3
C7—C8—Cl1	119.23 (9)	H16A—C16—H16B	109.2
C10—C9—C8	119.03 (11)	O3—C17—C16	106.63 (11)
C10—C9—H9	120.5	O3—C17—H17A	110.4
C8—C9—H9	120.5	C16—C17—H17A	110.4
C9—C10—C12	120.49 (11)	O3—C17—H17B	110.4
C9—C10—H10	119.8	C16—C17—H17B	110.4
C12—C10—H10	119.8	H17A—C17—H17B	108.6
C12—C11—C7	122.63 (11)	O2—B1—O1	119.71 (10)
C12—C11—Cl2	118.30 (9)	O2—B1—C1	118.96 (9)
C7—C11—Cl2	119.07 (9)	O1—B1—C1	121.33 (9)
C10—C12—C11	119.01 (12)	B1—O1—H1A	115.3 (13)
C10—C12—H12	120.5	B1—O2—H2A	113.1 (12)
C11—C12—H12	120.5	C14—O3—C17	109.77 (10)
			
C6—C1—C2—C3	−0.15 (16)	Cl1—C8—C9—C10	179.55 (9)
B1—C1—C2—C3	−178.97 (10)	C8—C9—C10—C12	0.09 (18)
C6—C1—C2—Cl3	−179.42 (8)	C8—C7—C11—C12	−0.07 (17)
B1—C1—C2—Cl3	1.76 (14)	C6—C7—C11—C12	178.17 (11)
C1—C2—C3—C13	−0.56 (17)	C8—C7—C11—Cl2	179.60 (8)
Cl3—C2—C3—C13	178.72 (9)	C6—C7—C11—Cl2	−2.15 (15)
C13—C5—C6—C1	−0.63 (18)	C9—C10—C12—C11	−0.26 (19)
C13—C5—C6—C7	177.91 (11)	C7—C11—C12—C10	0.25 (18)
C2—C1—C6—C5	0.74 (16)	Cl2—C11—C12—C10	−179.42 (10)
B1—C1—C6—C5	179.56 (10)	C2—C3—C13—C5	0.69 (18)
C2—C1—C6—C7	−177.77 (9)	C6—C5—C13—C3	−0.11 (19)
B1—C1—C6—C7	1.05 (15)	O3—C14—C15—C16	33.84 (13)
C5—C6—C7—C11	−90.69 (13)	C14—C15—C16—C17	−35.53 (14)
C1—C6—C7—C11	87.86 (13)	C15—C16—C17—O3	25.23 (15)
C5—C6—C7—C8	87.44 (14)	C2—C1—B1—O2	−111.66 (12)
C1—C6—C7—C8	−94.00 (13)	C6—C1—B1—O2	69.59 (14)
C11—C7—C8—C9	−0.11 (16)	C2—C1—B1—O1	69.17 (14)
C6—C7—C8—C9	−178.35 (10)	C6—C1—B1—O1	−109.58 (12)
C11—C7—C8—Cl1	−179.56 (8)	C15—C14—O3—C17	−18.86 (14)
C6—C7—C8—Cl1	2.21 (14)	C16—C17—O3—C14	−4.30 (16)
C7—C8—C9—C10	0.10 (17)		

### Hydrogen-bond geometry (Å, º)

**Table:**

*D*—H···*A*	*D*—H	H···*A*	*D*···*A*	*D*—H···*A*
O1—H1*A*···O3	0.82 (1)	1.84 (1)	2.6475 (12)	169 (2)
O2—H2*A*···O1^i^	0.84 (1)	1.96 (1)	2.7997 (12)	175 (2)

Symmetry code: (i) −*x*+2, −*y*+1, −*z*+1.
